# Moving Time

**Published:** 1869-04

**Authors:** 


### MOVING TIME.

The first of May is moving time in New York, and it will soon be upon us. Some like moving—variety is charming; if one is moving up, it has its satisfactions! For example, if you are moving from another's house into your own; if you are moving into more commodious quarters; if you are moving into a better, more cheery and more airy location; if you are moving out of a boarding-house and are going to keep house yourself, even if it be in a shanty; if you are moving from below Fourteenth street, nearer to the Park; if you are moving from the rivers to the ridge, that is, getting nearer Fifth Avenue; for it is an undisputed fact, that Fifth Avenue in its whole extent is the highest, driest, cheeriest and healthiest street in New York; yes, on the globe! No wonder that persons of taste and wealth and culture are willing to pay a hundred thousand dollars for a house in that splendid thoroughfare. We do not wonder that the movings referred to have their pleasures and their quiet satisfactions. But there are movings down, as well as movings up. The downers are in the following category: to have to move into a side street; to have to go nearer to the rivers; from a high stoop to an English basement; from your own attic or cellar, to somebody else's boarding-house; from a city home to Brooklyn or Jersey City or Staten Island, or over Harlem River; or to the country. We would rather save the difference in rent by living low; by living on bread and molasses and cold water. Living in the country is unwise for a New Yorker; you are always in a hurry or in a stew, for fear you will be "left." Your only leisure moment is from Saturday afternoon until Sunday night; every potatoe and apple costs you half a dollar a piece; what you do get to eat has to be lugged from town; no servant of any account will go to the country; they've got more sense. Go to the country! splendid! A race for life daily, to get to the cars, through noisome streets and dirty alleys; and then an hour of sweltering heat, of suffocating dust, of fiery, glary, dazzling, blinding sunshine; and such a rattle and a clatter of car wheels; such a dreadful screeching of locomotive whistles; such a thunderation and inky darkness and stifling smoke in the tunnels; and all this for the luxury of sweet milk that could not be sold in New York the day before; and for fresh eggs, laid in lime-water for three months. Living on the banks of the Hudson! It sounds splendid; but I saw a man last summer in the interior of Germany, in a little town on the second floor of a three-story house, on a street about twenty feet wide, and a sidewalk two feet; he had a splendid country seat of his own on the Hudson, lying idle; he had left it years ago and did not want to see it for years to come, because he liked the apartments he occupied better, was happier, enjoyed everything more. Where is the man on the banks of the Hudson for two or three years, who would not gladly sell out to-morrow? We know men who have been for years trying to sell out; and if they who live on the banks of the Hudson are sick and tired of it, how much more are they who live on the Jersey Sand Flats; among the agues of Long Island, and the musquitoes of Staten Island? The country is a good place for farmers and persons of elegant leisure. We don't deny that we ourselves like now and then to go into the country for about twenty-four hours; but longer than that we would rather be excused, unless travelling on horseback, or camping out, far away from human habitations. Old Gotham is good enough for us, and we never want to leave it "for long."

There are very many nice and clever people in the country, and the city is the better for having their pure blood and sturdy constitutions grafted into her own wavering strength of body and brain; but we have been speaking of it in connection with the annual moving process—that it is a "moving down," to have to go out of the city into the country on account of the relative rents. Those who have been born and bred in the country, and make farming and grazing and fruit culture their business, are the bone and sinew of the land, and are the only creators of money. Why, really we have forgotten where we left off, and have wandered away from our subject. We meant at first to say something practical and healthful to those who move about, changing their habitations in the city.

When you leave a house, leave it empty, swept and clean, fit for any decent person to occupy. Wherever soapsuds or whitewash can be properly applied, let them be abundantly used. But it is very likely you will have to move into a house that looks like a pig-pen; the cellar, especially, will be filled with all sorts of unclean things, decaying vegetables, bones, skins, old shoes, greasy tubs and the like; the cob-webs will show you that the walls have not been swept within the memory of man; the closets and pantries with their shelving will show grease and soiled spots and stains of every color and description; these will tell you exactly what your predecessors were. Let your first and greatest care be given to the cellar; move nothing into it; move every movable thing out of it; let every grating and door and window be kept wide open, from daylight until bed time; let the walls and floor be swept as clean as a stiff broom can do it. Let this sweeping and airing be repeated every day for a week; then whitewash the ceilings and the walls two or three coats with lime that has never been slacked; then scatter several pounds of copperas around the walls and in the corners. All this care should be taken, because if the air of the cellar is bad, it is constantly ascending into the building, your parlors, your chambers and your halls, so that you are breathing it day and night; for, as New York houses are constructed, the noisome, close air of the cellar has but little chance of escape except through the habitable part of the house; hence the utmost pains should be taken to keep the cellars of our dwellings sweet and clean. There ought to be no cellar under any human habitation.

